# Adolescent THC Exposure Causes Enduring Prefrontal Cortical Disruption of GABAergic Inhibition and Dysregulation of Sub-Cortical Dopamine Function

**DOI:** 10.1038/s41598-017-11645-8

**Published:** 2017-09-12

**Authors:** Justine Renard, Hanna J. Szkudlarek, Cecilia P. Kramar, Christina E. L. Jobson, Kyra Moura, Walter J. Rushlow, Steven R. Laviolette

**Affiliations:** 1 Dept. of Anatomy and Cell Biology & Dentistry, University of Western Ontario, London, Ontario, N6A 5C1 Canada; 2 Dept. of Psychiatry, Schulich School of Medicine & Dentistry, University of Western Ontario, London, Ontario, N6A 5C1 Canada

## Abstract

Chronic adolescent marijuana use has been linked to the later development of psychiatric diseases such as schizophrenia. GABAergic hypofunction in the prefrontal cortex (PFC) is a cardinal pathological feature of schizophrenia and may be a mechanism by which the PFC loses its ability to regulate sub-cortical dopamine (DA) resulting in schizophrenia-like neuropsychopathology. In the present study, we exposed adolescent rats to Δ-9-tetra-hydrocannabinol (THC), the psychoactive component in marijuana. At adulthood, we characterized the functionality of PFC GABAergic neurotransmission and its regulation of sub-cortical DA function using molecular, behavioral and *in-vivo* electrophysiological analyses. Our findings revealed a persistent attenuation of PFC GABAergic function combined with a hyperactive neuronal state in PFC neurons and associated disruptions in cortical gamma oscillatory activity. These PFC abnormalities were accompanied by hyperactive DAergic neuronal activity in the ventral tegmental area (VTA) and behavioral and cognitive abnormalities similar to those observed in psychiatric disorders. Remarkably, these neuronal and behavioral effects were reversed by pharmacological activation of GABA_A_ receptors in the PFC. Together, these results identify a mechanistic link between dysregulated frontal cortical GABAergic inhibition and sub-cortical DAergic dysregulation, characteristic of well-established neuropsychiatric endophenotypes.

## Introduction

Marijuana is the most widely used illicit drug among adolescents. Although it is still a matter of debate, there is evidence suggesting that chronic exposure to the primary psychoactive phytochemical in cannabis, tetrahydrocannabinol (THC), increases the long-term risk of psychiatric diseases, including schizophrenia^1–5^. The risk is higher depending upon age of onset of chronic marijuana use (e.g. before 17) and relative THC concentration. Nevertheless, only a minority of cannabis users may be prone to develop psychiatric diseases. It is therefore likely that both environmental factors and genetic predisposition play a role in this causal association. Animal models are useful tools for investigating the long-term behavioral effects of cannabis exposure during adolescence. We have previously demonstrated that chronic THC exposure during adolescent neurodevelopment leads to persistent abnormalities in adulthood resembling schizophrenia, involving neuropathological molecular adaptations in the prefrontal cortex (PFC), concomitant with a hyperactive sub-cortical dopamine (DA) system^6^. These effects are of particular clinical relevance given the increasing levels of THC in current strains of marijuana^7^. Nevertheless, the precise neurobiological mechanisms underlying the long-term neuropsychiatric effects of adolescent THC exposure are still unknown.

Adolescence is a highly vulnerable period for brain development, during which the PFC undergoes massive functional remodeling. These cortical remodeling processes include refinement of intrinsic GABAergic function and changes in the excitatory–inhibitory neuronal balance, essential for synchronized cortical activity, regulation of sub-cortical emotional processing centers, and maturation of normal adult behavior and cognition^8, 9^. Importantly, due to their strategic location on local PFC GABAergic circuits, cannabinoid type 1 receptors (CB1R) play a key role in prefrontal maturational processes, maintaining the balance of excitatory/inhibitory neuronal activity and associated cortical oscillatory states^10–12^. Consequently, neurodevelopmental impairment of PFC CB1R signaling and associated GABAergic function has the potential to cause long-term pathological prefrontal disinhibition, impaired synchronized cortical activity states and psychopathological deficits in PFC-dependent function^8, 13^.

The onset of schizophrenia during adolescent neurodevelopment^14, 15^ is linked to impairments in PFC GABAergic signaling. For example, numerous studies demonstrate cortical reductions in the GABA synthesizing enzyme, glutamic acid decarboxylase-67 (GAD67) corresponding to a loss of GABAergic parvalbumin (PV) interneurons in post-mortem frontal cortical tissues from schizophrenia patients^16–24^. Altered expression levels of select prefrontal cortical GABA_A_ receptor subunits have also been observed in schizophrenia, characterized by increased expression patterns of GABA_A_ receptor α2 subunits and decreased levels of the γ2 and δ subunits^25–27^. It has been proposed that this cortical GABAergic dysfunction may underlie disturbances in gamma-band synchronized neuronal activity associated with pathological emotional and cognitive deficits observed in schizophrenia^28^. For example, cortical gamma oscillation power has been reported to be increased in schizophrenia^29, 30^, a phenomena that is thought to be associated with the positive symptoms of schizophrenia, including hallucinations^31, 32^. In addition, decreased activity of inhibitory PFC GABAergic neurons and a resulting dysregulation of PFC pyramidal neuron activity and associated gamma oscillatory states, may be associated with sub-cortical DAergic dysregulation, and related affective and cognitive disturbances in schizophrenia^33^. For example, knockdown of the α3 GABA_A_ receptor subunit causes a hyper-DAergic, schizophrenia-like phenotype linked to hyperactive DAergic activity states in the ventral tegmental area (VTA)^34^ consistent with the effects of chronic THC exposure during adolescent neurodevelopment^6^. Furthermore, GABA_A_ receptor blockade or direct electrical stimulation of the PFC induces schizophrenia-like behaviors and increases sub-cortical DA release^35–37^.

Using a validated rodent model of adolescent THC exposure, we hypothesized that neurodevelopmental exposure to THC would lead to a molecular, neuronal and behavioral phenotype resembling schizophrenia due to a loss in GABAergic regulation of the PFC and associated sub-cortical DAergic activity patterns. We report that adolescent THC exposure induces a long-term loss of GABAergic inhibition within the PFC that persists into adulthood. This phenotype was characterized by dysregulated γ oscillatory activity, downregulation of GABAergic protein markers, increased medial PFC (mPFC) output neuron activity, hyperactive sub-cortical DAergic activity and a range of cognitive and affective abnormalities resembling to those observed in psychiatric diseases such as schizophrenia. Remarkably, pharmacological activation of GABA_A_ receptors directly in the mPFC, which shares functional-anatomical properties with the human dorsolateral PFC^38, 39^, reversed these deficits, demonstrating that aberrant PFC-mesolimbic connectivity following adolescent THC exposure is a critical neuropathological mechanism underlying an increased risk for later adulthood schizophrenia-like abnormalities.

## Results

### THC exposure during adolescence reduces expression levels of GAD67 in the adult mPFC

**Figure 1:**
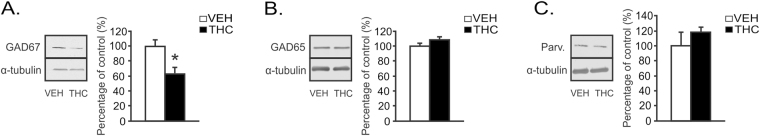
Long-term effects of chronic THC exposure during adolescence on mPFC GABAergic markers. (**A**) Representative western blot for GAD67 expression in the mPFC (left). A significant decrease in GAD67 expression is observed between adolescent VEH and adolescent THC pretreated rats. **(B**) Representative western blot for GAD65 expression in the mPFC (left). No significant changes in GAD65 were found between groups. **(C**) Representative western blot for parvalbumin (PV) expression in the mPFC (left). No significant changes in PV were found between groups. n = 8 rats, t-tests; *Indicated *p* < 0.05. Error bars represent the standard error of the means (SEMs). Western blots for GAD 67, GAD65 and parvalbumin levels are shown in Supplementary Figure 1.

Schizophrenia is associated with lower levels of GAD67 in the dorsolateral PFC^17^. Using Western Blotting (Fig. 1), we analyzed mPFC protein expression levels of GAD67 comparing adult rats treated with VEH or THC during adolescence. Western blot analysis of GAD 67 revealed a dramatic decrease in adolescent THC *vs*. VEH-treated rats (t_(6)_ = 2.44; *p* < 0.05; Fig. 1A). This demonstrated strong reductions in mPFC GABAergic levels following adolescent THC exposure, consistent with schizophrenia neuropsychopathology. Conversely, we did not observe changes in mPFC protein expression levels of either GAD65 or parvalbumin (PV) in adolescent THC *vs*. VEH-treated rats (*t*_(6)_ = −0.33; *p* > 0.05 and t_(6)_ = −0.33; *p* > 0.05, respectively; Fig. 1B,C).

### Adolescent THC exposure increases mPFC putative pyramidal neuron firing and bursting Rates

**Figure 2:**
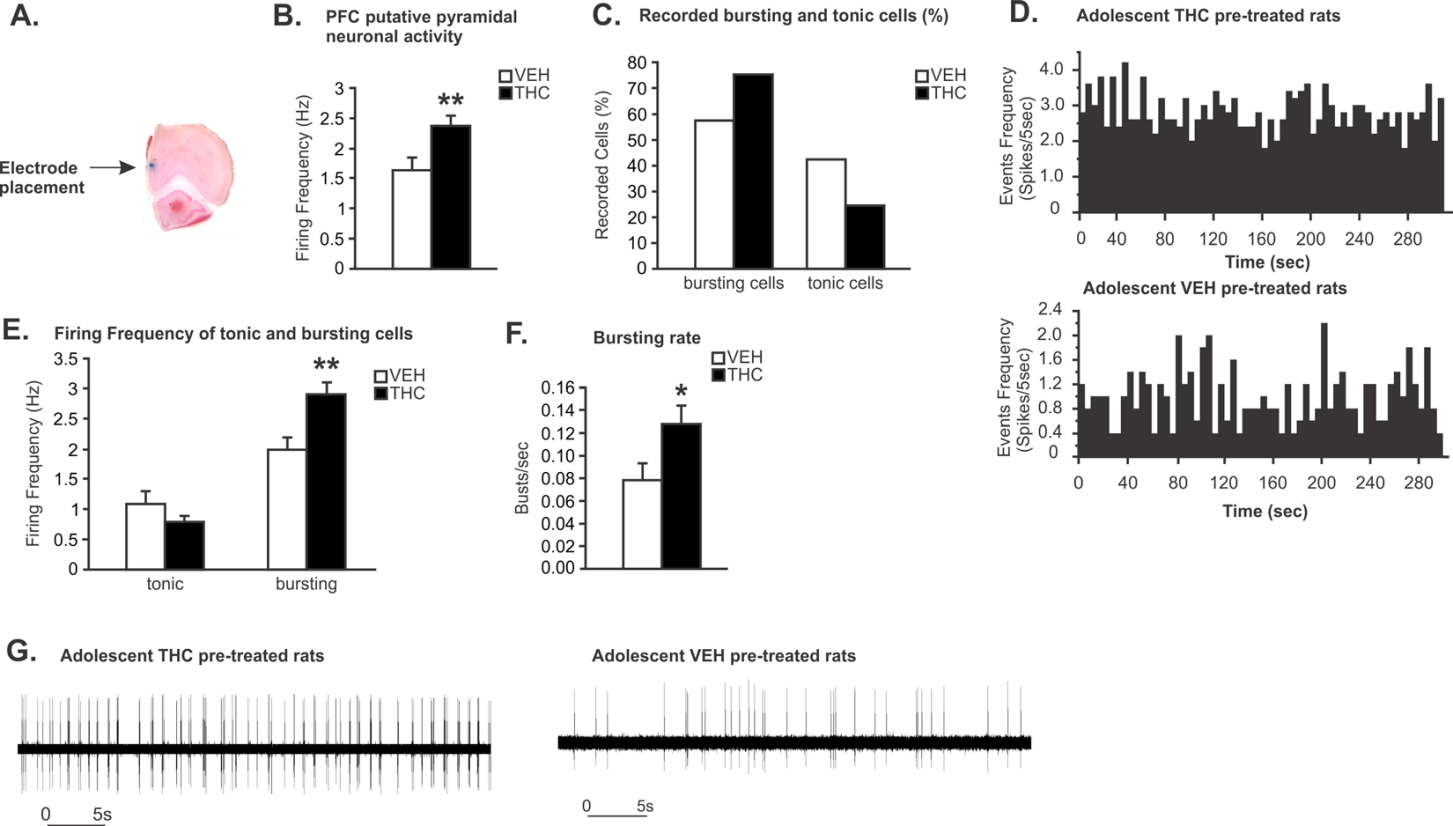
Long-term effects of chronic THC exposure during adolescence on spontaneous mPFC putative pyramidal neuron activity. (**A**) Microphotograph of a representative mPFC neuronal recording placement. **(B)** Adolescent THC pretreated rats displayed increasing spontaneous PFC putative pyramidal neuronal firing frequency. **(C)** Greater proportion of bursting neurons was observed in adolescent THC exposed rats when compared to VEH controls (75.41% *vs*. 57.58%). **(D)** Representative rastergrams showing spontaneous activity of putative PFC pyramidal neurons in THC (top) *vs*. VEH pretreated rats (bottom). **(E)** Firing frequencies of bursting cells, not tonic cells, were significantly higher in adolescent THC exposed rats when compared to VEH controls. **(F)** In the bursting cells population, the bursting rate of putative pyramidal neurons of adolescent THC-exposed rats was significantly higher than in VEH controls. **(G)** Representative examples showing bursting activity of putative PFC pyramidal neurons in THC (left) *vs*. VEH pretreated rats (right). Two-tailed t-tests; **indicated p < 0.01; *indicated p < 0.05. Error bars represent the standard error of the means (SEMs).

We next evaluated the long-term effects of adolescent THC exposure on adult spontaneous mPFC putative pyramidal neuron activity parameters (See Methods). A microphotograph of a representative mPFC neuronal recording placement is shown in Fig. 2A. In THC treated rats, statistical analysis revealed that THC-exposed rats displayed significantly increased mPFC putative pyramidal neuronal firing frequencies *(t*_(219)_ = 3.131; *p* < 0.01; Fig. 2B) relative to VEH controls. In addition, a greater proportion of bursting neurons was observed in THC *vs*. VEH exposed controls (75.41% *vs*. 57.58%; Fig. 2C). Representative rastergrams showing spontaneous activity of mPFC putative pyramidal neurons in THC *vs*. VEH pretreated rats are presented in Fig. 2D. When comparing spontaneous firing frequencies of mPFC putative pyramidal neurons displaying tonic *vs*. bursting patterns, firing rates of bursting, but not tonic cells, were significantly higher in THC vs. VEH exposed controls (*t*_(147)_ = −2.862; *p* < 0.01; Fig. 2E). Finally, in the subpopulation of bursting neurons, spontaneous bursting rates of putative pyramidal neurons were significantly higher in THC *vs*. VEH exposed controls (*t*_(147)_ = 2.055; *p* < 0.05; Fig. 2G). Recording traces comparing bursting patterns in putative PFC pyramidal neurons in THC *vs*. VEH control rats are presented in Fig. 2G.

### Adolescent THC treatment disrupts spontaneous adult cortical gamma oscillations

**Figure 3:**
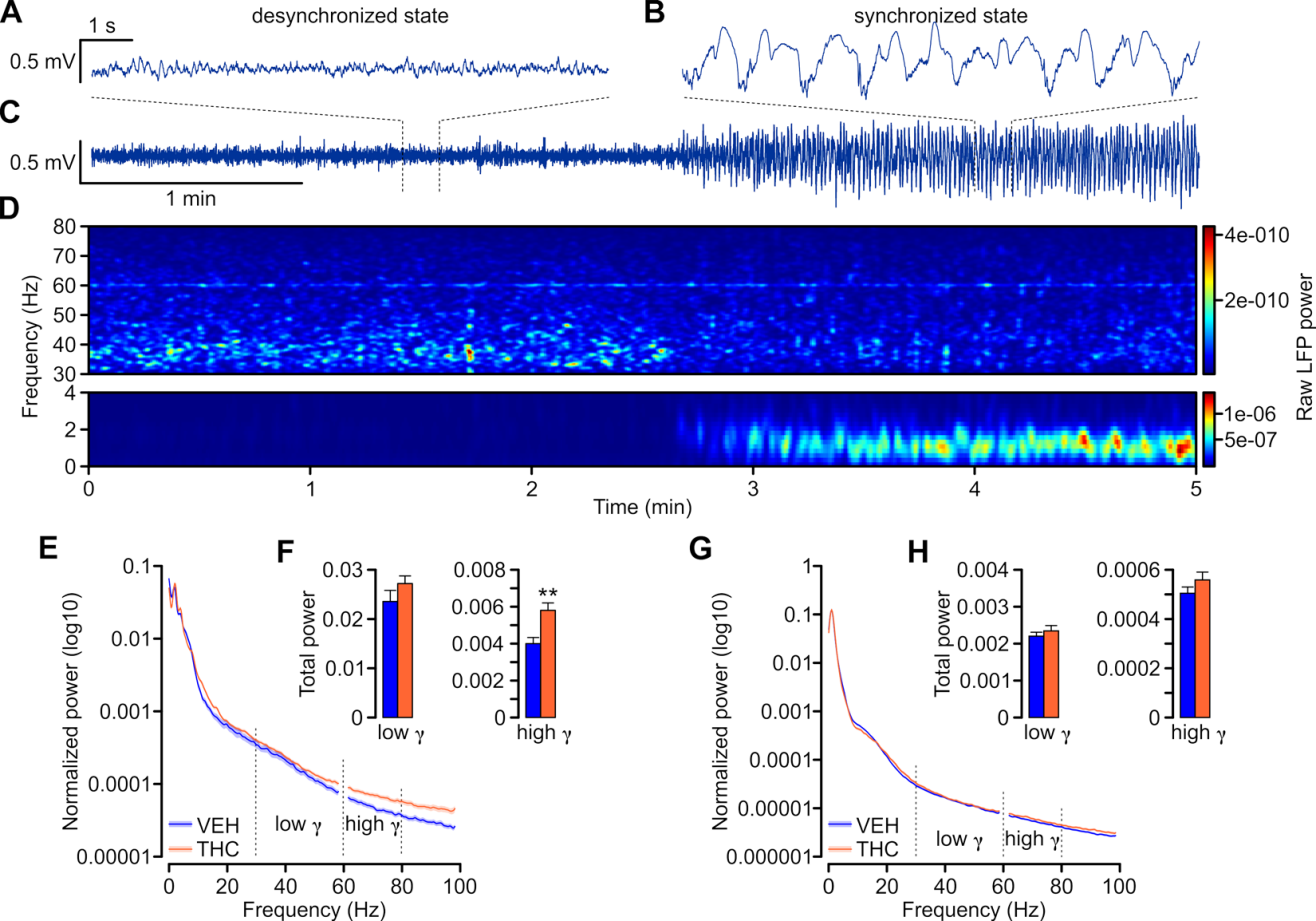
Adolescent THC-treatment lead to increased high gamma (61–80 Hz) power in the mPFC of adult rat. (**A**–**C**) Example recording traces from PFC of urethane anesthetized rat showing different cortical states. Desynchronized state **(A)** was characterized by small-amplitude fast oscillations while synchronized state **(B)** by large-amplitude slow oscillations. **(C)** Five-minute recording showing spontaneous alternation from the desynchronized to synchronized state. **(D)** Spectrogram calculated for a five-minute recording presented in **C** showing the temporal changes in the power at different frequencies. Note that upon transition from desynchronized to synchronized state gamma power decreased while the slow delta oscillation gradually emerged. The power values are color-coded as indicated on the right-hand side insets. A peak at around 60 Hz reflect power line frequency and the LFP power values for frequencies between 59–61 Hz were excluded from further analysis. **(E**,**G)** Average normalized power spectra corresponding to prefrontal LFP of VEH- (blue) and THC-treated (orange) rats in desynchronized **(E)** and synchronized **(G)** states. Note the increased power of high-gamma band (61–80 Hz) in THC-treated rats. **(F**,**H)** Bar graphs summarizing the average total power of the low- and high-gamma calculated for desynchronized **(F)** and synchronized **(H)** states of VEH (blue) and THC-treated (orange) rats. The high-gamma of THC-treated rats was significantly more powerful than VEH. Two-tailed t-tests; **indicated p < 0.01. Error bars represent the standard error of the means (SEMs).

Local field potential (LFP) recordings in the mPFC were performed in urethane anesthetized rats and were typically characterized by occurrence of slow, high-voltage fluctuations (>0.5 mV; Fig. 3B) corresponding to a synchronized cortical state. Occasionally, epochs of desynchronized states occurred, characterized by small-voltage (<0.5 mV) and fast oscillations (Fig. 3A). The strength of the oscillations was assessed by calculating power spectral densities (PSD; window length = 2 s; shift = 0.5 s; Fig. 3D) over a 1-min long recording epoch, averaging PSDs over time and subsequently normalizing the averaged PSD to the total power (normalized total power = 1; values for frequencies between 59 and 61 Hz were excluded from calculations to avoid contamination with 60 Hz noise from power line).

During the desynchronized states the gamma oscillations were approximately 10 times more powerful than during the synchronized states. The total gamma power, calculated as a sum of power values for frequencies between 30–80 Hz of THC-treated rats did not differ from VEH controls (desynchronized LFPs: VEH = 0.0275 ± 0.0028, THC = 0.0330 ± 0.0018, *t*_(96)_ = −1.6467; *p* > 0.05) synchronized LFPs: VEH = 0.0027 ± 0.0001; THC = 0.0029 ± 0.0001; *t*_(172)_ = −0.9325; *p* > 0.05). However, the high-gamma oscillations (61–80 Hz) of the desynchronized state were significantly increased in THC-treated rats when compared with VEH-treated rats (VEH = 0.0040 ± 0.0003; THC = 0.0058 ± 0.0004; *t*_(96)_ = −3.1411; *p* < 0.01 Fig. 3F). These data suggest impairments of the intracortical inhibitory feedback within the PFC circuit and are consistent with the increased firing of putative pyramidal cells (Fig. 2) and decreased expression of GAD67 in THC-treated rats (Fig. 1A).

### Intra-mPFC GABA_A_ receptor activation reverses deficits in short term memory induced by adolescent THC exposure

**Figure 4:**
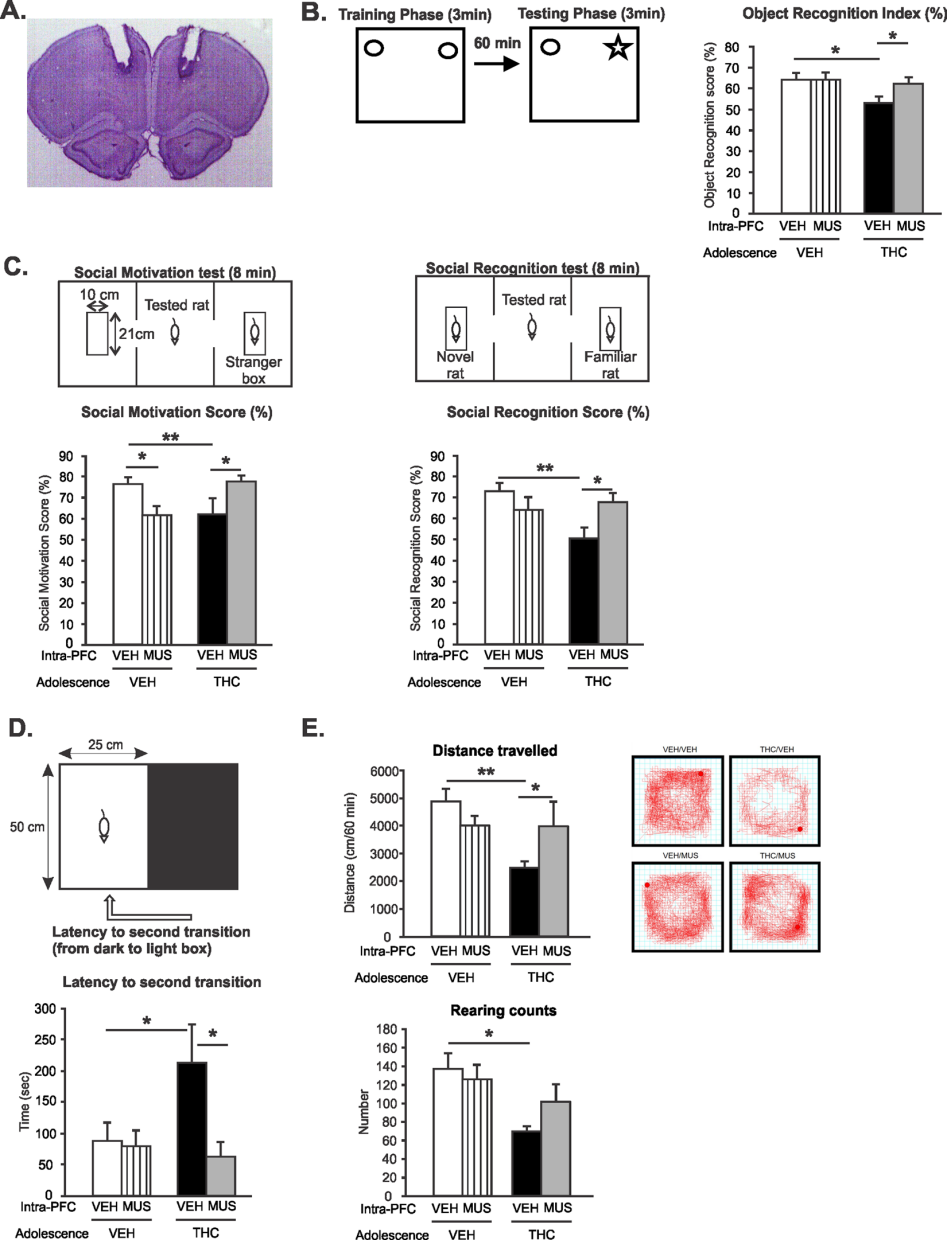
Effects of Intra-mPFC MUS on adolescent THC-induced behavioral abnormalities. (**A)** Microphotograph of a representative Intra-mPFC injector placement. **(B)** THC exposure during adolescence induced short-term memory deficits in the object recognition task. Intra-mPFC MUS treatment reversed adolescent THC-induced short-term memory deficits relative to intra-mPFC VEH controls. **(C)** THC exposure during adolescence induced lower social motivation (left) and social cognition (right) index in the social interaction task. Intra-mPFC MUS treatment reversed adolescent THC-induced social motivation (left) and social cognition (right) deficits relative to intra-mPFC VEH controls. **(D)** THC exposure during adolescence increased anxiety levels in the light dark box test. Intra-mPFC MUS reduced adolescent THC-induced anxiety relative to intra-mPFC VEH controls. **(E)** THC exposure during adolescence decreased locomotion (top) and rearing counts (bottom) in the open field test. Intra-mPFC MUS reduced adolescent THC-induced hypolocomotion relative to intra-mPFC VEH controls (top) but had no effects on rearing counts. Anova 2 factors **indicated p < 0.01; *indicated p < 0.05. Error bars represent the standard error of the means (SEMs).

We next mechanistically examined the potential role of PFC GABA_A_ receptor transmission by microinfusing the selective GABA_A_ agonist, muscimol (MUS; 500 ng/0.5 μl) directly into the mPFC to determine if pharmacologically activating GABA_A_ receptors may reverse behavioral abnormalities associated with adolescent THC exposure. A microphotograph of a representative intra-mPFC injector placement is showed in Fig. 4A. Memory deficits are core features of schizophrenia and are thought to be associated with abnormal prefrontal GABA function^13^. Therefore, we measured short-term memory performance of adult rats pretreated with VEH or THC during adolescence using the object recognition task. Two-way ANOVA revealed a significant main effect of treatment on recognition index (F_(1,34)_ = 4.717; *p* < 0.05). *Post hoc* analysis revealed that THC exposure during adolescence induced short-term memory deficits when compared to adolescent VEH pretreated rats (*p* < 0.05); Fig. 4B). Intra-mPFC MUS treatment reversed adolescent THC-induced short-term memory deficits relative to intra-mPFC VEH controls (*p* < 0.05; Fig. 4B) while having no effects in adolescent VEH pretreated rats (*p* > 0.05). Thus, intra-mPFC MUS infusion restored cognitive performances in adolescent THC pretreated rats.

### Intra-mPFC GABA_A_ receptor activation reverses deficits in social cognition induced by adolescent THC exposure

Affective impairment and social cognition represent negative symptoms of schizophrenia^40–43^. In addition, the PFC plays a critical role in regulating social cognition in both humans and rodents^43–46^. Therefore, we next compared social motivation/cognition performances in THC *vs*. VEH treated groups using the social novelty preference test. During the social motivation test, two-way ANOVA revealed a significant interaction between adolescent exposure and PFC treatment factors (F_(1,41)_ = 9.062; *p* < 0.01). *Post hoc* analysis revealed that rats exposed to THC during adolescence displayed a lower social motivation index when compared to VEH controls (*p* < 0.05); Fig. 4C left). Intra-mPFC GABA_A_ receptor activation impaired social motivation performances in VEH treated rats (*p* < 0.05; Fig. 4C left) while reversing adolescent THC-induced social cognition deficits relative to controls (*p* < 0.05; Fig. 4C left).

During the social cognition test, two-way ANOVA revealed a significant interaction between adolescent exposure and PFC treatment factors (F_(1,41)_ = 6.67; *p* < 0.05). *Post hoc* analysis revealed that rats exposed to THC during adolescence displayed a lower social memory index when compared to VEH controls (*p* < 0.01); Fig. 4C right). Intra-mPFC GABA_A_ receptor activation reversed adolescent THC-induced social cognition deficits relative to intra-mPFC VEH controls (*p* < 0.05; Fig. 4C right) while having no effects in adolescent VEH pretreated rats (*p* > 0.05).

### **Intra-**m**PFC GABA**_**A**_**receptor activation reduces increased anxiety levels following adolescent THC exposure**

Next, the effects of intra-mPFC MUS microinfusion on anxiety levels were examined in THC *vs*. VEH treated rats using the light-dark box anxiety test (Fig. 4D). The latencies to first transition (i.e. moving from the light to dark environment) and second transition (i.e. moving from the dark to light environment) were recorded and compared between the different drug treatment conditions. Two-way ANOVA analysis of the latency to first transition revealed non-significant effects of either adolescent exposure or adult intra-mPFC treatment or interaction between factors, indicating that all groups of rats displayed similar entry latency times (*data not shown*). However, two-way ANOVA analysis of the latency to second transition indicated a slight yet non-significant effect of adult intra-mPFC treatment (F_(1,39)_ = 3.68; *p* = 0.062). *Post hoc* comparisons showed that in adolescent THC pretreated rats the latency to re-emerge from the dark compartment was increased when compared to adolescent VEH pretreated rats (*p* < 0.05; Fig. 4D, bottom), demonstrating increased anxiety in adolescent THC pretreated rats, consistent with previous findings^6^. Intra-mPFC MUS reduced the latency to reemerge from the dark compartment in adolescent THC pretreated rats relative to intra-mPFC VEH controls (*p* < 0.05; Fig. 4D, bottom) while having no effects on adolescent VEH pretreated rats (*p* > 0.05). Thus, intra-mPFC MUS infusion decreased anxiety levels in adolescent THC pretreated rats.

### **Intra-**m**PFC GABA**_**A**_**receptor activation reduces deficits in exploratory behavior induced by adolescent THC exposure**

Adolescent THC exposure has been shown to decrease motivational exploratory behaviors^6^. Therefore, we next tested if intra-mPFC GABA_A_ receptor activation may reverse this deficit. Two-way ANOVA revealed a significant effect of adolescent treatment on total distance travelled (F_(1,40)_ = 6.145; *p* < 0.05). *Post hoc* analysis revealed that adolescent THC pretreated rats showed decreased locomotion when compared to VEH controls (*p* < 0.01; Fig. 4E, top). However, intra-mPFC MUS restored locomotor activity levels in THC treated rats relative to VEH controls (*p* < 0.05; Fig. 4E, top) while having no effects on adolescent VEH pretreated rats (*p* > 0.05). Examples of typical activity plots for adolescent VEH or THC-intra-mPFC or MUS treated rats are presented in Fig. 4E. Analysis of rearing counts revealed a significant main effect of adolescent treatment (F_(1,40)_ = 9.82; *p* < 0.01). *Post hoc* analysis demonstrated that THC treated rats exhibited significantly less rearing behavior *vs*. VEH controls (*p* < 0.01; Fig. 4E, bottom). However, intra-mPFC MUS had no effects in either of the adolescent VEH or THC pretreated groups (*p* > 0.05; Fig. 4E, bottom). Thus, intra-mPFC MUS infusion restored exploratory motor behavior in adolescent THC pretreated rats.

### Intra-mPFC GABA_A_ receptor activation reverses sub-cortical DAergic hyperactivity induced by adolescent THC exposure

**Figure 5:**
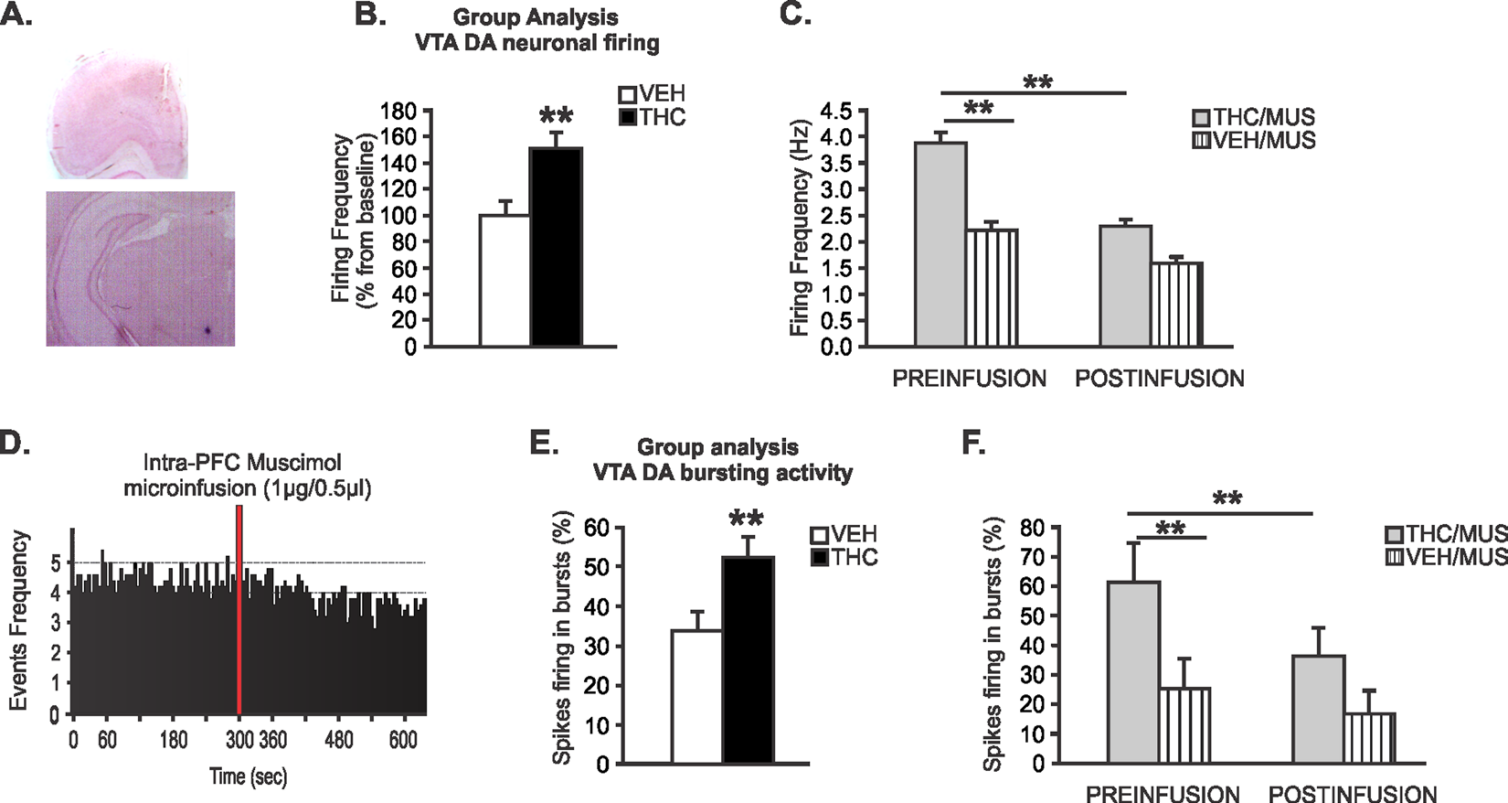
Effects of Intra-mPFC MUS on adolescent THC-induced sub-cortical hyperdopaminergia. (**A**) microphotograph of a representative mPFC microsyringe and VTA neuronal recording placements. **(B)** Increased VTA putative DA neuronal firing frequency in adolescent THC-pretreated rats relative to adolescent VEH-pretreated rats. **(C)** Before intra-mPFC MUS microinfusion, sub-population of VTA putative DA neurons firing frequency in adolescent THC pretreated rats were significantly increased compared to adolescent VEH pretreated rats. Intra-mPFC MUS reduced firing frequency of VTA putative DA in adolescent THC-pretreated rats relative to adolescent VEH-pretreated rats. **(D)** Representative rastergram showing the spontaneous activity of 1 putative DA neuron treated with intra-mPFC muscimol in adolescent THC pretreated rat. **(E)** Increased VTA putative DA neurons bursting levels in adolescent THC *vs*. VEH pretreated rats. **(F)** Before intra-mPFC MUS microinfusion, sub-population of VTA putative DA neurons spikes firing in bursts in adolescent THC pretreated rats were significantly increased compared to adolescent VEH pretreated rats. Intra-mPFC MUS reduced spikes firing in bursts of VTA putative DA in adolescent THC-pretreated rats relative to adolescent VEH-pretreated rats. Two-way repeated measures ANOVA or Two-tailed t-tests; **indicated p < 0.01. Error bars represent the standard error of the means (SEMs).

Given our previous findings of significantly reduced GABAergic PFC GAD67 activity and PFC neuronal hyperactivity in following adolescent THC exposure, we hypothesized that pharmacologically restoring PFC GABA_A_ receptor tone may reverse the hyperactive VTA DA neuron phenotype induced by adolescent THC exposure. Using *in vivo*, single-unit VTA neuronal recordings in putative DAergic VTA neurons (see Methods) we next examined the effects of intra-mPFC MUS infusions on putative VTA DAergic neuronal frequency and bursting rates. Microphotographs of a representative PFC injector placement and VTA neuronal recording site are shown in Fig. 5A. Collective analysis of baseline firing frequency levels before mPFC micro-infusions of either MUS or VEH showed increased neuronal firing frequency in adolescent THC-pretreated rats (n = 40 neurons) relative to adolescent VEH-pretreated rats (n = 45 neurons) (t_(83)_ = 3.15, *p* < 0.01, Fig. 5B). For analysis purposes, a neuron was considered to have changed its firing rate if there was a minimum of 10% difference in frequency rate from baseline. Using this previously reported criterion^47^, qualitative analyses revealed that in VEH pretreated rats, intra-mPFC MUS microinfusions caused mixed effects on firing rates with 10% of putative DA neurons showing increased firing, 40% decreased, and 50% unchanged. In THC pretreated rats that received intra-mPFC MUS, 12% of putative DA neurons showed increased in firing frequency, 46% decreased, and 42% were unchanged. One-way ANOVA comparing pre *vs*. post-infusion firing rates in sub-populations of putative DA neurons showing decreased firing following mPFC MUS, revealed a significant effect of recording epoch (F_(1,22)_ = 13.002; *p* < 0.01) on firing rates. *Post hoc* comparisons revealed that before mPFC MUS infusions, VTA putative DA neuron baseline frequency in THC pretreated rats was significantly increased relative to VEH controls (*p* < 0.01; Fig. 5C). In addition, in adolescent THC treated, but not VEH controls, the firing frequency of VTA putative DA neurons was significantly reduced by mPFC MUS infusions (*p* < 0.01; Fig. 5C). Thus, pharmacological activation of GABA_A_ transmission in the mPFC was able to effectively reverse the hyper-DAergic phenotype induced by adolescent THC exposure. A representative rastergram showing the spontaneous activity of a putative DA neuron treated with intra-mPFC muscimol in a THC pretreated rat is presented in Fig. 5D.

Similar to VTA putative DA neuron firing frequency rates, collective analysis of spontaneous baseline levels of putative DA neuron bursting activity comparing THC *vs*. VEH control neurons demonstrated increased bursting rates specifically in THC-treated rats (t_(83)_ = −2.86; *p* < 0.01; Fig. 5E). One-way ANOVA comparing the sub-populations of neurons from THC and VEH rats showing intra-mPFC MUS-induced decreased bursting levels showed a significant effect of recording epoch (F_(1,11)_ = 13.12; *p* < 0.01) on bursting rate levels. Similar to firing frequency, *post hoc* comparisons revealed that baseline bursting levels of VTA putative DA neurons recorded in THC treated rats were significantly increased relative to VEH controls (*p* < 0.05; Fig. 5F). In addition, in adolescent THC, but not VEH treated rats, intra-mPFC MUS significantly reduced bursting rates relative to pre-infusion levels (*p* < 0.01; Fig. 5F). Thus, intra-mPFC activation of GABA_A_ receptor transmission was able to effectively reverse the hyper-bursting VTA putative DA neuron phenotype induced by adolescent THC exposure. mPFC VEH microinfusions had no effects on either DA neuron firing frequency or bursting levels in either VEH or THC treated rats (data not shown). These results suggest that hyperexcitability of VTA DA neurons induced by adolescent exposure to THC can be attenuated by pharmacologically restoring the inhibitory balance within the mPFC.

## Discussion

In the present study, we demonstrate that adolescent THC exposure induces persistent prefrontal GABA hypofunction in adulthood characterized by reduced expression levels of the GABAergic marker GAD67, increased spontaneous PFC pyramidal neuron bursting and firing rates and potentiated high gamma power oscillatory activity. Furthermore, these cortical neuronal abnormalities occurred in concert with a sub-cortical hyperDAergic neuronal phenotype in the VTA. Finally, adolescent THC exposure induced cognitive and affective disturbances characterized by impairments in short-term memory, social motivation and cognition, higher anxiety levels and decreased motivation. Remarkably, these behavioral impairments were reversed by pharmacological activation of GABA_A_ receptors in the adult mPFC which concomitantly, reversed sub-cortical hyperDAergic activity in the VTA. Together, these results identify a mechanistic link between dysregulated frontal cortical GABAergic inhibition and sub-cortical DAergic dysregulation, characteristic of well-established schizophrenia endophenotypes.

First, the observed decrease of GAD67 in the adult mPFC following adolescent THC exposure is consistent with schizophrenia-related cortical neuropathology. Decreased cortical GABA function is one of the most reliable neuropathological markers observed in post-mortem analyses of the schizophrenic brain^48^. Both mRNA and protein expression of GAD67 are reduced in the dorsolateral PFC of individuals with schizophrenia^16, 19, 21–23^ suggesting a reduction in cortical GABA synthesis and release. Furthermore, consistent with previous findings in schizophrenia, we observed no PFC GAD65 alterations^49^. Our results are consistent with previous studies showing GAD67 reductions in selective parvalbumin (PV)- and CCK-containing PFC interneuron populations following adolescent THC exposure^50^. Similar to previous reports, we did not find any THC-induced changes in total PFC PV levels, suggesting that, despite a deficit in cortical GABAergic synthesis, overall population levels of PV interneurons are likely not attenuated^50^.

Consistent with a loss of cortical GABAergic inhibition, we observed a significant potentiation in both firing and bursting rates of putative mPFC pyramidal neurons, demonstrating a persistent dysregulation of cortical excitatory/inhibitory balance following adolescent THC exposure. This increase in firing frequency was concomitant with a general increase in the proportion of mPFC neurons displaying bursting patterns of activity, suggesting a general shift from tonic to bursting neuronal activity states. In the PFC, pyramidal burst-firing generally indicates greater signal to noise ratio, which in turn can pathologically amplify the strength of selective signals entering the cortex. Moreover, altered burst-firing patterns of pyramidal PFC neurons can be indicators of dysregulated pyramidal cell function due to changes in the integration of multiple neuronal inputs^51^, which may underlie cognitive, motivational and affective deficits observed in psychiatric disorders. Importantly, cannabinoid transmission strongly modulates PFC pyramidal neuron encoding of associative emotional memories. For example, acute overstimulation of CB1R transmission in the PFC causes profound misattribution of emotional salience cues measured in associative fear memory paradigms, both by increasing the firing frequency and associative bursting activity of PFC neurons^52, 53^. Thus, adolescent THC exposure induces a PFC neuronal phenotype consistent with deficits in the ability to appropriately integrate competing cortical inputs in terms of their affective and/or attentional relevance, consistent with the profound behavioral abnormalities observed following adolescent THC exposure.

Consistent with our observation of mPFC neuronal hyperactivity and loss of GABAergic inhibitory tone, we observed a significant and persistent potentiation in gamma-band oscillatory activity states in the adult mPFC, following adolescent THC exposure. In the healthy brain, the interplay between GABA interneurons and excitatory pyramidal cells is critical for the generation of synchronous gamma oscillations^54^. This cortical oscillatory activity represents a fundamental mechanism to enable the normal functioning of distributed cortical networks and supports a variety of higher order information processing necessary for normal cognition, learning and memory. Considerable evidence demonstrates that dysregulated PFC gamma oscillations are a critical neuropathological feature of schizophrenia and may serve as a neurodevelopmental signature for the onset of first-episode schizophrenia^55^. For example, affective and social cognition disturbances are associated with abnormal patterns of PFC gamma activity in schizophrenia^56, 57^. In addition, hallucinations and reality distortion have been correlated with increased cortical gamma power in schizophrenia^58, 59^. High (>60 Hz) gamma-band oscillatory dysregulation may contribute to schizophrenia-related perceptual and cognitive deficits^60–62^. While numerous studies have reported reduced evoked levels of cortical gamma band activity, other studies have found that schizophrenia patients demonstrate potentiated spontaneous gamma band oscillatory patterns in the PFC and during working memory tasks^63, 64^, believed to be related to a loss of cortical GABAergic inhibitory mechanisms.

At the cellular level, CB1R transmission plays a role in the control of neuronal network gamma oscillations. For example, acute CB1R stimulation alters neuronal network oscillations in the gamma-frequency ranges^65–67^ in both hippocampus and cortex. Similarly, 5 days of exposure to a synthetic CB1R agonist during early adolescence in rats impaired the modulation of cortical oscillatory activity evoked by hippocampal stimulation^8^. Thus, acute or chronic CB1R activation can alter network oscillations in the mammalian PFC. Given the critical role of CB1R signaling in frontal cortical maturation^8^ and maintenance of normal oscillatory activity, the present findings provide further evidence for the critical role of neurodevelopmental CB1R signaling in the maturation of inhibitory cortical networks.

Given our evidence for a profound hypo-GABAergic PFC phenotype following adolescent THC exposure and studies linking downregulation of cortical GABA_A_ receptor expression levels with schizophrenia-related deficits^68^, we examined the potential mechanistic role of mPFC GABA_A_ receptor transmission by pharmacologically activating mPFC GABA_A_ receptors. Remarkably, we found that direct mPFC GABA_A_ receptor activation reversed adolescent THC-induced impairments in short-term memory as well as motivational and anxiety-related abnormalities in adult rats, while having no effects on adolescent VEH pretreated rats. Thus, it appears that under normal conditions, such as in adolescent VEH pretreated rats, enhancing mPFC GABAergic transmission has no effects on cognitive performance. In contrast, under pathological conditions involving hypo-GABAergic function, such as in adolescent THC pretreated rats, activation of mPFC GABA_A_ transmission can reverse these deficits. Therefore, similar to observations in schizophrenia^35, 69, 70^ decreasing prefrontal GABA_A_ transmission and disinhibition of the mPFC can disrupt cognitive and emotional function. However, these cognitive and emotional impairments can be restored by increasing prefrontal GABA_A_ transmission. Similarly, it has been shown that mPFC inactivation via infusion of the GABA_A_ receptor agonist muscimol, did not affect working and reference memories in rats tested in the radial-arm maze task. In contrast, mPFC disinhibition via infusions of the GABA_A_ receptor antagonist bicuculline impairs both working and reference memory^71^ and impairs performance of PFC-independent cognitive tasks, suggesting that hyperactivity of the frontal lobes may interfere with cognitive/mnemonic processes mediated by downstream structures^71^. Thus, it is possible that in adolescent THC pretreated rats, memory processes may be disrupted by disinhibitory increases in mPFC activity, consistent with the mPFC glutamatergic hyperactivity that we found in these animals. Since the mPFC sends excitatory projections to the VTA^72^, it is possible that mPFC disinhibition-induced cognitive impairments may be driven by aberrant patterns of excitatory outflow to sub-cortical, mesolimbic structures. Therefore, although decreasing prefrontal GABA_A_ transmission/disinhibition of the mPFC impaired cognitive performance, inhibition of mPFC neural activity with GABA agonists had no effect on cognitive performance in adolescent VEH pretreated rats but might selectively inhibit mPFC hyperactivity in adolescent THC pretreated rats.

Interestingly, we found that intra-mPFC GABA_A_ receptor activation reversed social behavior deficits in adolescent THC pretreated rats, but caused impairments in VEH control rats. Similar results have been observed in previous studies. For example, intra-mPFC muscimol was shown to block social familiarity-induced reductions in anxiety in healthy rats^73^. Thus, under normal conditions, enhancing mPFC GABAergic transmission appears to suppress social motivation. However, under pathological conditions involving hypo-GABAergic function, such as those induced by adolescent THC exposure, increasing mPFC GABAergic tone may reverse social deficits and cognition, as demonstrated by the present findings, suggesting that the normal balance between excitation and inhibition within the prefrontal circuitry is crucial for the integration of socially relevant cues and social memory formation and that adolescent THC exposure may compromise normal social cognition by disturbing an intra-mPFC inhibitory GABAergic network.

Interestingly, clinical studies have demonstrated therapeutic effects of GABA-modulating drugs on schizophrenia-related symptoms, including cognitive deficits, hallucinations, agitation and anxiety^74, 75^. Moreover, increasing GABA_A_ receptor function in schizophrenia patients with chronic administration of a GABA_A_ α2/3-selective agonist was shown to reverse working memory deficits and cognitive control impairments, as well as normalizing gamma band power abnormalities associated with these symptoms^68^. To our knowledge, the present findings represent the first demonstration that a THC-induced neurodevelopmental cortical GABAergic deficit can be functionally reversed by direct pharmacological intervention in adulthood.

Increasing mPFC GABAergic tone not only rescued adolescent THC-induced affective/cognitive deficits, but also normalized VTA DAergic activity levels. Dysregulation of the mesocorticolimbic DA pathway is a cardinal neuropathological feature associated with schizophrenia-related symptoms. In terms of PFC-VTA interactions, the PFC sends dense glutamatergic, regulatory projections to VTA DA neurons^76^. Thus, we hypothesized that our observed sub-cortical DAergic hyperactive phenotype may be directly linked to hyperactive excitatory drive from the PFC. Consistent with this model, previous studies have reported that PFC GABA_A_ receptor antagonist infusions induced schizophrenia-like cognitive and affective disturbances in rodents and primates^35, 77^. Furthermore, the partial GABA_A_ receptor agonist imidazenil reverses methionine-induced schizophrenia-like deficits in rodents, including social interaction and sensorimotor gating impairments^78^. In addition, knockout of the GABA_A_ α3-subunit was shown to induce a hyper-DAergic state and sensorimotor gating deficits which could be reversed with antipsychotics^34^. Since tonic DA release is thought to be controlled by PFC GLUTergic afferents^79^, one possible mechanistic explanation is that direct activation of PFC GABA_A_ transmission within the PFC, may reverse PFC overdrive to sub-cortical DA targets and associated cognitive and affective deficits, as suggested by the present findings.

In summary, our results demonstrate that hypofunction of inhibitory PFC GABAergic neurotransmission plays a crucial role in the behavioral and neuronal effects observed following adolescent THC exposure. These findings improve our knowledge of the precise neurobiological mechanisms leading to the long-term deleterious effects of THC exposure during adolescence and the later risk of schizophrenia onset. More importantly, these findings demonstrate that THC-induced neurodevelopmental insults to the PFC-mesolimbic circuitry may be reversible by restoration of GABAergic cortical tone, even into adulthood.

## Methods

### Animals and housing

Adolescent Sprague-Dawley rats were obtained at postnatal day (PND) 28 from Charles River Laboratories (Quebec, Canada). At arrival, rats were pair housed under controlled conditions (12 h light/dark cycle, constant temperature, and humidity) with free access to food and water. All procedures were performed in accordance with Governmental and Institutional guidelines for appropriate rat care and experimentation. The experimental protocols were approved by the Canadian Council on Animal Care and the Animal Care Committee at Western University, Ontario.

### Adolescent THC administration

Rats treated with THC (Cayman Chemical) received twice daily injections of THC (2.5 mg/kg; Days 1–3; 5 mg/kg; Days 4–7; 10 mg/kg, Days 8–11). Control groups received the same injection schedule (volume adjusted per body weight) with vehicle (VEH). Increasing doses of THC were administered to counter the development of drug tolerance^80^. These doses of THC were chosen based on our and others previously published protocols^6, 50, 81–83^ and are known to produce long-term behavioral and molecular and neuronal effects in rats. Importantly, this escalating THC exposure protocol mimics a heavy use regiment of marijuana exposure, with the 2.5 mg/kg dose corresponding to approximately one cannabis cigarette, the 5 mg/kg dose corresponding to approximately two cannabis cigarettes, and the 10 mg/kg dose corresponding to approximately four cannabis cigarettes^50^. THC in ethanol was dissolved in cremophor and saline (1:1:18). Ethanol was then evaporated using nitrogen gas to remove it from the final THC solution. All injections were administered intraperitoneally (i.p). The adolescent exposure experiments began at PND 35. Experimental procedures were initiated following a 30 days drug-free period (at PND 75).

### Protein Expression Analyses

At adulthood (PND75), rats received an overdose of sodium pentobarbital (240 mg/kg, i.p., Euthanyl^TM^). Under deep anesthesia rats were decapitated and brains removed and frozen. Coronal sections (60 μm) containing the PFC were cut on a cryostat and slide mounted. Bilateral micro-punches of the mPFC, were obtained for protein isolation. The western blotting procedure was performed as described previously^84^. Primary antibody dilutions were as follows: α-tubulin (1:120 000; Sigma-Aldrich), GAD67 (1:1000; Cell Signaling Technology), GAD65 (1:200; Santa Cruz Biotechnology) and Parvalbumin (1:2000; Sigma-Aldrich). Species appropriate horseradish peroxidase (HRP)-conjugated secondary antibodies (Thermo Scientific) were all used at a dilution of 1:20 000.

### Neuronal Activity Recordings and Analysis

Extracellular single-unit electrophysiological recordings were performed *in vivo* in adult (>PND 75) VEH and THC pretreated rats during adolescence. The recordings were taken from either putative glutamatergic (GLUT) PFC neurons (cells/animals: VEH = 99/6, THC = 122/10) or dopaminergic (DA) VTA neurons (cells/animals: VEH = 45/24, THC = 40/20). Rats were anesthetized with urethane (1.4 g/kg, i.p., Sigma-Aldrich) and placed in a stereotaxic frame with body temperature maintained at 37 °C. A scalp incision was made and a hole was drilled in the skull overlaying the targeted structure at the following coordinates: mPFC: AP: +2.7 to +3.5 mm from bregma, L: ±0.8 to ±1 mm, DV: −2.5 to −4 mm from the dural surface; VTA: AP: −5.2 mm from bregma, L: ±0.8 to ±1 mm, DV: −6.5 to −9 mm from the dural surface. Recordings were taken with glass microelectrodes (average impedance of 6–10 MΩ) filled with 2 M sodium acetate solution containing 2% pontamine sky blue (Sigma-Aldrich). A bone screw was placed over the cerebellum and was connected with the return of the headstage and served as a reference electrode. Extracellular signals were amplified (×5000) using a MultiClamp 700B amplifier (Molecular Devices), digitized at 25 kHz and recorded on the computer using a Digidata 1440 A and pClamp software (Molecular Devices). The wideband signal of PFC recordings was fed to two channels of the digitizer and filtered to obtain single unit recordings (band pass between 0.3 and 3 kHz) and local field potentials (LFPs; low pass at 0.3 kHz). For the VTA electrophysiology only unit data were recorded.

Putative mPFC pyramidal cells were identified based on established criteria^85^: (1) firing frequency < 10 Hz, (2) waveform shape, and (3) action potential duration > 2.5 ms. Cells exhibiting 3 consecutive spikes with inter-spike intervals < 45 ms were classified as burst-firing cells^85^. The electrophysiological properties of spontaneously active pyramidal neurons were sampled in the mPFC by making vertical passes of the electrode through the pyramidal cell body region. These tracks were made in a predefined pattern, with each track separated by 200 µm. After an individual putative pyramidal neuron was isolated, its spontaneous activity was recorded for 5 min. Two parameters of activity were sampled, the basal firing rate and the bursting rate.

VTA DA neurons were identified according to well established electrophysiological features^86^: (1) action potential width > 2.5 ms, (2) spontaneous firing rate between 2–5 Hz, (3) a triphasic waveform consisting of a notch on the rising phase followed by a delayed after potential, and (4) a single irregular or burst firing pattern. For intra-mPFC microinfusions of VEH or Muscimol (MUS, 1 μg/0.5 μl) a 10 μl Hamilton syringe was slowly lowered into the mPFC using the same stereotaxic coordinates as described above. The firing frequencies of collective VTA putative DA neurons from adolescent THC pretreated rats before microinfusions of VEH or muscimol into the mPFC were averaged and normalized to the average firing frequency of collective VTA neurons from adolescent VEH pretreated rats. The response patterns of isolated VTA neurons to microinfusion of VEH or MUS into the mPFC were determined by comparing the neuronal frequency rates between the 5 min preinfusion *vs*. postinfusion recording epochs. We also analyzed the proportion of DA neuronal spikes fired in burst mode. The onset of a burst was defined as the occurrence of two consecutive spikes with an interspike interval of 80 ms^86^. The percentage of burst spikes was calculated by dividing the number of spikes occurring in bursts by the total number of spikes occurring in the same period of time. We sampled a total of n = 85 VTA DA neurons (Adolescent VEH pretreated-Intra mPFC VEH group; n = 15 cells in 7 rats; Adolescent VEH pretreated- Intra-mPFC MUS group; n = 30 cells in 17 rats; Adolescent THC pretreated- Intra mPFC VEH group; n = 14 cells in 7 rats; Adolescent THC pretreated- Intra mPFC MUS group; n = 26 cells in 13 rats).

LFP signals were analyzed using NeuroExplorer (Nex Technologies). First, the signals were decimated to 1 kHz, and lowpass filtered (IIR Butterworth filter at 170 Hz; filter order set to 3). Subsequently a spectrogram function was used to calculate the power of oscillations at frequencies between 0–100 Hz (window length 2 s; shift 0.5 s). One minute long recording epochs were used for estimating the average power spectrum distributions. Epochs were selected such as either the desynchronized (relatively small signal amplitude devoid of slow oscillations) or synchronized (relatively large signal amplitude with slow oscillations present) cortical state could be easily distinguished. Power values for a given frequency were averaged over time of the recording epoch and normalized so that the sum of all power spectrum values equals 1. The total power was calculated by adding all the power values at frequencies between 0–59 and 61–100 Hz. Power values at 60 ± 1 Hz were excluded from all the calculations. Gamma band was defined as frequency between 30–80 Hz and subcategorized into low gamma (30–59 Hz) and high gamma (61–80 Hz). Statistics are based on 98 recordings of desynchronized state (VEH = 37, THC = 61) and 174 recordings of synchronized state (VEH = 107, THC = 67). Every recording was taken at different electrode location throughout mPFC.

For histological analyses the recording electrodes positions were marked with an iontophoretic deposit of pontamine sky blue dye (−20 mA, continuous current for 15 min). Histological analysis was performed as described previously^47^. No mPFC cells were recorded outside the anatomical boundaries of the mPFC as defined by Paxinos and Watson (2007). One recorded rat was excluded from electrophysiological data analysis because its cells were recorded outside the anatomical boundaries of the VTA as defined by Paxinos and Watson (2007).

### Surgical Procedures

At adulthood (PND) 75, rats were anesthetized with an intraperitoneal injection of ketamine (80 mg/mL, Vetoquinol)-xylazine (6 mg/kg, Bayer) mixture. To minimize pain and inflammation, meloxicam (1 mg/kg; s.c., Boehringer Ingelheim) was administered before and after surgeries. Rats were positioned in a Kopf stereotaxic device for cannulae implantation. Stainless steel guide cannulae (22-gauge) were implanted bilaterally into the mPFC with the coordinates (15**°** angle, in mm from bregma): AP: +2.9 L: ±1.9 mm: DV: −3 mm from the dural surface. The guide cannulae were secured into position by jeweler’s screws and dental acrylic cement. Rats were singly housed post-surgery and behavioral tests were initiated after one week of recovery.

### Intra-mPFC Microinfusions

Intra-mPFC microinfusions of either vehicle (VEH, NaCl 0.9%) or the selective GABA_A_ receptor agonist, muscimol (500 ng/0.5 µl, Sigma-Aldrich, diluted in NaCl 0.9%) were performed immediately before behavioral experiments. A total volume of 0.5 μl per hemisphere was delivered via a 28 gauge injector over a period of 1 min. Microinjectors were left in place for an additional 1 min following infusions to ensure adequate diffusion from the tip.

### Behavioral Testing

#### Object recognition

Rats were tested using the object recognition task as described previously^87^. This task evaluates the ability of the rat to discriminate between the familiarity of previously encountered objects; normal rats typically spend more time exploring a novel object than a familiar object. The test sessions consisted of two 3-min trials. During the first trial (T1 acquisition trial), each rat was placed in the center of an arena containing two identical objects placed in the far corners 15 cm from the side wall. After a delay of 60 min during which the rat was returned to its cage, and both objects were replaced (one by an identical copy, the other by a novel object in the same location), the rat was returned to the arena for the second trial (T2 test trial). Between rats, both the role (familiar or novel object) and the relative position of the two objects were randomly counterbalanced. Object exploration was considered when the head of the rat was facing the object or the rat was touching or sniffing the object. Times spent in exploration were videotaped with a video-tracking system (ANY-maze; Stoelting) and analyzed by an experimenter blind to the treatment conditions. Exploration times were recorded and used to calculate object recognition index [time spent with novel object/total time exploring both objects] *100. The final number of rats in each group was as follows: Adolescent VEH-Intra-mPFC VEH (VEH/VEH) group, n = 10; Adolescent VEH-Intra-mPFC MUS (VEH/MUS) group, n = 9; Adolescent THC-Intra-mPFC VEH (THC/VEH) group, n = 9; Adolescent THC-Intra-mPFC MUS (THC/MUS) group, n = 10.

#### Social motivation and social cognition

Rats were tested using a social interaction procedure as described previously^6^. Briefly, this task evaluates 2 distinguishable aspects of social behavior: (1) social affiliation/motivation and (2) social recognition memory. Rats were habituated to the test arena for 13 min, 24 h before testing. Testing consisted of 2 successive 8-min phases. During the first phase, we analyzed social motivation, that is, the propensity to spend time with an unfamiliar male rat (stranger rat) enclosed in a small wire cage compared with time spent with an identical but empty cage. During the second phase, occurring just after the first one, we analyzed social recognition, that is, the propensity to spend time with a novel unfamiliar rat (novel stranger) rather than with the familiar stranger rat (encountered during the first phase). The locations of stranger *vs*. novel rats in the left *vs*. right side chambers were counterbalanced between trials. Times spent in exploration were videotaped with a video-tracking system (ANY-maze; Stoelting) and analyzed by an experimenter blind to the treatment conditions. After each test, chambers and cages were cleaned with 50% ethanol to avoid olfactory cue bias. Exploration times were recorded and used to calculate a social motivation or cognition index [time spent with stranger (or novel stranger)/total time exploring both rats] *100. The final number of rats in each group was as follows: Adolescent VEH-Intra-mPFC VEH (VEH/VEH) group, n = 12; Adolescent VEH-Intra-mPFC MUS (VEH/MUS) group, n = 11; Adolescent THC-Intra-mPFC VEH (THC/VEH) group, n = 12; Adolescent THC-Intra-mPFC MUS (THC/MUS) group, n = 10.

#### Light dark box

This test is based upon a rat’s natural aversion to bright environments and attributes greater time spent in an illuminated environment as reflecting lower anxiety levels. The test was performed as previously described^6^. At the start of the experiment, a rat was placed in the center of the lighted box with its head facing the wall opposite the door and was allowed to freely explore both compartments for a period of 8 min. A zone entry was considered to have begun when the rat placed all 4 paws in that zone. Experiments were videotaped with a video-tracking system (ANY-maze;Stoelting) and analyzed by an experimenter blind to treatment conditions. Behaviors analyzed included latency time to leave the dark box and enter the light box (latency to second transition), which is thought to be the most reliable indicator of anxiety-like behavior and is sensitive to both anxiogenic and anxiolytic treatments^88^. The final number of rats in each group was as follows: Adolescent VEH-Intra-mPFC VEH (VEH/VEH) group, n = 9; Adolescent VEH-Intra-mPFC MUS (VEH/MUS) group, n = 12; Adolescent THC-Intra-mPFC VEH (THC/VEH) group, n = 12; Adolescent THC-Intra-mPFC MUS (THC/MUS) group, n = 10.

#### Open field test

Rats were placed in an automated open field activity chamber (San Diego Instruments, San Diego, CA, USA) for 60 min. Total distance travelled and vertical counts were recorded and analyzed. The final number of rats in each group was as follows: Adolescent VEH-Intra-mPFC VEH (VEH/VEH) group, n = 11; Adolescent VEH-Intra-mPFC MUS (VEH/MUS) group, n = 12; Adolescent THC-Intra-mPFC VEH (THC/VEH) group, n = 12; Adolescent THC-Intra-mPFC MUS (THC/MUS) group, n = 9.

### Statistical analysis

The data were analyzed using *t*-tests, one or two-way ANOVA. *Post hoc* analyses were calculated using Fisher’s LSD. Densitometry values for Western blots were acquired with Kodak digital analysis software and analyzed with *t*-tests.

### Data availability

The authors declare that the data supporting the findings of this study are available from the corresponding author on reasonable request.

## Electronic supplementary material

Supplementary Figure 1
