# Supplementary material for: Adolescent THC Exposure Causes Enduring Prefrontal Cortical Disruption of GABAergic Inhibition and Dysregulation of Sub-Cortical Dopamine Function
Source: Sci Rep. 2017 Sep 12;7:11420. doi: 10.1038/s41598-017-11645-8 (PMC5595795; doi:10.1038/s41598-017-11645-8)
Supplement: Supplementary file 1 — Supplementary Figure 1 [file 41598_2017_11645_MOESM1_ESM.doc]

**Supplementary data**

**Adolescent THC Exposure Causes Enduring Prefrontal Cortical Disruption of GABAergic Inhibition and Dysregulation of Sub-Cortical Dopamine Function**

Justine Renard^1^, Hanna J. Szkudlarek^1^, Cecilia P. Kramar^1^, Christina E.L. Jobson^1^, Kyra Moura^1^, Walter J. Rushlow^1,2^ & Steven R. Laviolette^1,2*^

^1^Dept. of Anatomy and Cell Biology & ^2^Dept. of Psychiatry, Schulich School of Medicine & Dentistry, University of Western Ontario, London, Ontario

CANADA N6A 5C1

***To whom correspondence should be addressed**: Steven R. Laviolette, Ph.D. Dept. of Anatomy & Cell Biology, Schulich School of Medicine & Dentistry, University of Western Ontario, London, Ontario. Canada. N6A 5C1. Phone: 1-519-661-2111. e-mail: steven.laviolette@schulich.uwo.ca

**Supplementary Figure 1.**

**
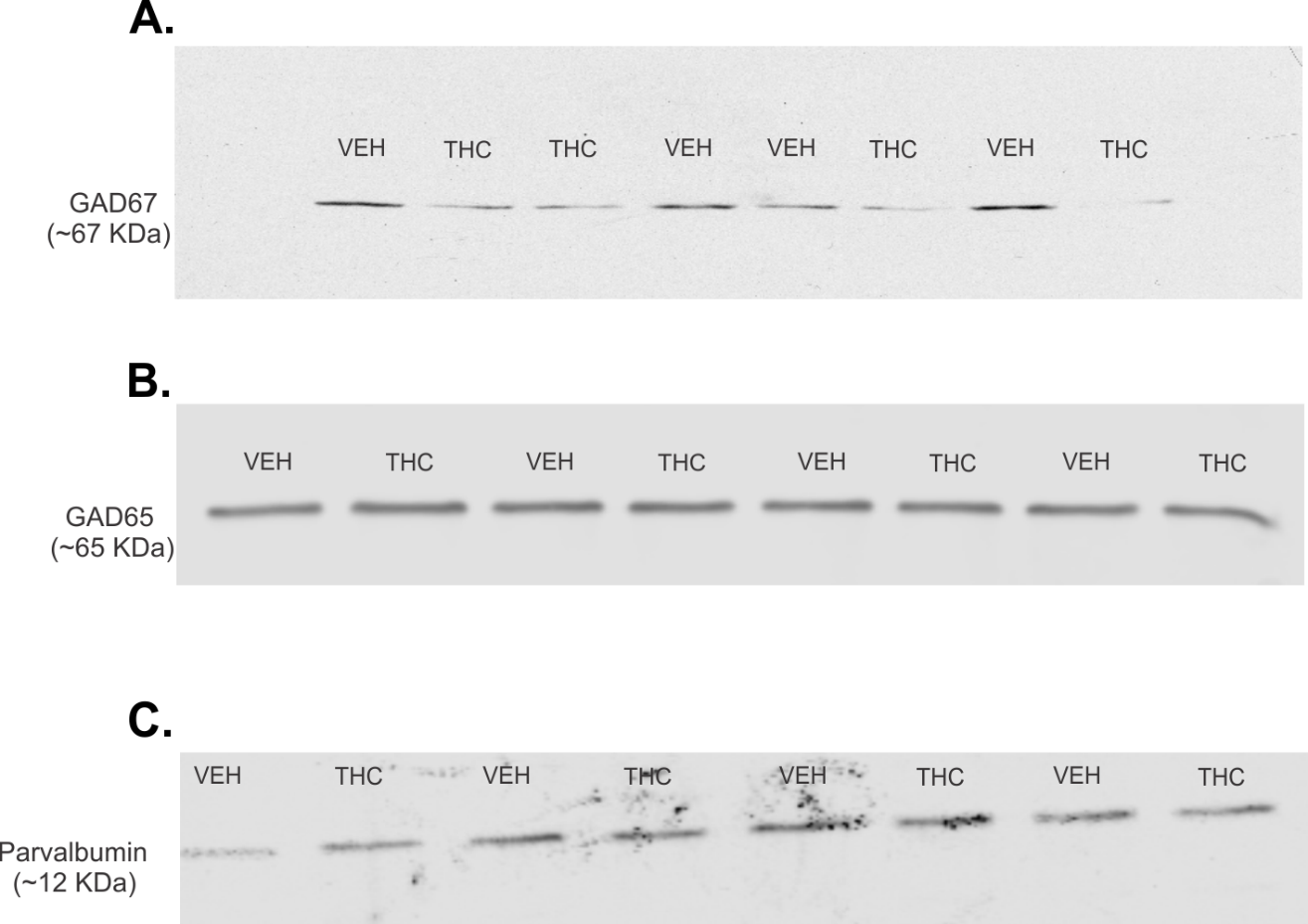
**

Western Blots for **(A)** GAD67, **(B)** GAD65, **(C)** parvalbumin (PV) levels in the mPFC; n= 8 rats (4 VEH and 4 THC)
